# Delivering preventive, predictive and personalised cancer medicine for renal cell carcinoma: the challenge of tumour heterogeneity

**DOI:** 10.1007/s13167-011-0137-3

**Published:** 2011-12-22

**Authors:** Rosalie Fisher, James Larkin, Charles Swanton

**Affiliations:** 1 Department of Medicine, Royal Marsden Hospital, London, UK; 2 Cancer Research UK London Research Institute, Translational Cancer Therapeutics Laboratory, 44 Lincoln's Inn Fields, London WC2A 3LY, UK

**Keywords:** Renal cell carcinoma, Heterogeneity, Personalised medicine, Predictive biomarkers

## Abstract

Recent years have seen major advances in the management of metastatic renal cell carcinoma (mRCC). The tyrosine kinase and mammalian target of rapamycin inhibitors have resulted in disease control and improved survival for many patients with mRCC, but they have not led to preventive, predictive or personalised medicine (PPPM). Failure to achieve this rests ultimately with inadequate knowledge of tissue and molecular heterogeneity; discovery of these drugs was based upon identification of pathogenic molecular pathways in RCC, but research into molecular factors which underpin drug response, resistance and selection of therapy for individual patients has lagged well behind clinical trials of drug development. This review will provide an overview of the development of targeted drug therapies for mRCC, will discuss the challenges which currently impede the delivery of PPPM, including identification of biomarkers, drug resistance and molecular heterogeneity, and will propose research methodologies and technologies required to overcome these obstacles.

## Introduction

Renal cell carcinoma is a relatively rare cancer in which prognosis is highly individual

Renal cell carcinoma (RCC) is an epithelial neoplasm arising from the parenchyma of the kidney, which accounts for 95% of renal neoplasms, and 3% of adult malignancies [1]. RCC is a relatively rare cancer, with an incidence of 60,000 cases in the European Union in 2006, but is associated with a high mortality rate; in the same year, there were 26,000 deaths due to this disease [2].

The prognosis in RCC has traditionally been thought to be influenced by tumour stage, nuclear grade and histologic tumour necrosis [3]. Those patients with disease confined to the kidney and regional lymph nodes are treated with nephrectomy (partial or radical) with curative intent. However, approximately one third of patients have metastases at the time of diagnosis [4], and a similar proportion develop metastatic disease within 5 years of follow up [5]. Common secondary sites include lymph nodes, lung and bone.

### Metastatic RCC is incurable

Metastatic RCC (mRCC) is incurable, and the aim of therapy for patients with advanced disease is to control the disease burden for as long as possible, thus ameliorating the patient's symptoms and improving quality of life, and prolonging overall survival time. Nephrectomy is still considered standard treatment for those patients who have a good performance status and a limited burden of metastatic disease, based on the results of two randomised studies which found a survival benefit for patients treated with nephrectomy and cytokine therapy, compared with cytokine therapy alone [6]. Historically, patients with mRCC have had extremely limited systemic treatment options and poor 5 year survival rates. Hormone therapy and chemotherapy produce response rates of 10% or less [7,8]. Cytokine therapy, including interferon alfa and high dose interleukin-2, may benefit a small proportion of patients, resulting in response rates of 10-20%, and a modest survival benefit of several months over non-immunotherapy controls [9,10]. A small number of patients may be cured with high dose interleukin-2 therapy. Despite the recent SELECT trial demonstrating a response rate of 29% to interleukin-2 [11], there are still no established criteria to select those patients who will benefit from immunotherapy, and these treatments have been associated with substantial toxicity. Thus, an accurate risk-benefit analysis for an individual patient is difficult.

### Shifted focus of drug development

In the last decade, drug development in oncology has shifted its focus from cytotoxic treatments toward biological therapies. The use of 'targeted' therapies is dependent on the identification of biological pathways that selectively confer a growth and/or survival advantage to the cancer cell. There are many examples of drugs which attempt to exploit the underlying biology of the tumour, including trastuzumab, used in Her-2 amplified breast cancer [12,13], the tyrosine kinase inhibitors imatinib for chronic myeloid leukaemia [14], and erlotinib and gefitinib in non-small cell lung cancer [15,16]. More recently, breakthroughs have occurred in two refractory tumours with the development of vemurafenib for *BRAF*-mutant melanoma [17] and crizotanib in patients with non-small cell lung tumours with rearrangement of the *ALK *gene [18]. Arguably, however, renal cell cancer is the solid tumour type that has enjoyed the most success from a targeted approach to therapy, and has the most number of biological agents available for clinical use. Six agents are now approved for mRCC, which target pro-angiogenic and proliferative pathways; the small molecule tyrosine kinase inhibitors sunitinib, sorafenib, and pazopanib, the monoclonal antibody bevacizumab, and the mammalian target of rapamycin (mTOR) inhibitors temsirolimus and everolimus. As a result, the prognosis for patients with mRCC has improved dramatically, and clinicians hope that mRCC may yet become a 'chronic disease' [19].

### RCC is characterised by much heterogeneity

Despite these advances, mRCC is a diverse disease with much clinical, pathological and molecular heterogeneity. This argues strongly for an individualised approach to therapy, but a number of obstacles stand in the way of preventive, predictive and personalised medicine for this condition. This review will discuss the pathological and molecular subtypes of RCC, the heterogeneity in clinical course and the role of systemic therapy in this context, and propose mechanisms by which tailored therapy for patients might be achieved.

## Pathological and molecular classification of RCC

The 2004 WHO classification system identifies distinct histological subtypes of RCC [20]; the major subtypes are clear cell, papillary types 1 and 2, chromophobe, and collecting duct cancers. Translocation and medullary tumours and mucinous tubular and spindle cell carcinomas are rare entities. The various subtypes are associated with diverse clinical outcomes, and distinguishing between them may provide a useful indication of prognosis, and guide to appropriate therapy, for an individual patient.

### Clear cell RCC

Clear cell RCC accounts for approximately 75% of malignant kidney tumours, and 90% of RCCs that metastasise [21]. So called 'conventional' clear cell RCCs are recognised histologically by clear cell cytoplasm; morphological variants include granular and sarcomatoid carcinomas. Clear cell RCC can be diagnosed from hematoxylin and eosin-stained microscopy, but they are also associated with a typical immunohistochemical pattern with positivity for vimentin, cytokeratin, and CA-IX [21,22]. Genetically, clear cell RCC is characterised by loss of DNA in the short arm of chromosome 3 (3p), which has been shown to occur in 79-90% of cases, as detected by FISH [23]. This region contains the Von Hippel-Lindau (*VHL*) gene, a tumour suppressor thought primarily responsible in the pathogenesis of hereditary and sporadic clear cell RCC. Inactivation of *VHL*, due to somatic mutation, or hypermethylation, occurs in 100% of familial RCCs as part of VHL disease, and in up to 80% of sporadic clear cell tumours [24,25]. The VHL protein (pVHL) regulates hypoxia-inducible factor (HIF) by ubiquitin-mediated destruction [26,27] and in the absence of functional pVHL, HIF activates a number of hypoxiaresponse genes such as vascular endothelial growth factor (VEGF), erythropoietin (Epo), platelet derived growth factor (PDGF), TFG-α and -β, all of which are associated with tumour angiogenesis and growth [28-30]. *HIF *gene expression is also increased by activation of mTOR, part of the complex PI3 kinase/Akt pathway, thereby contributing to angiogenesis [31], but this pathway also appears to be critical in promoting in cell growth and survival [32]. A point mutation in *mTOR*, R2505P, has been identified in renal cell carcinoma, and confers constitutive activation of mTOR signalling [33].

Epigenetic mechanisms, such as DNA methylation and modification of histone proteins, also control gene expression and have an important role in human cancers. Amongst other functions, both of these processes regulate chromatin structure, and are thereby implicated in transcriptional control [34]. Several studies have identified a number of chromatin modifying genes which appear to be closely related to this disease. The histone demethylases *JMJD1A *and *JMJD2B *have been recognised as transcriptional targets of HIF-1, and have increased expression in renal cancer cells which lack functional VHL protein [35,36]. A large-scale screen of coding exons of 3544 genes in 101 clear cell RCCs identified activating mutations in *SETD2 *and *JARID1C*, both encoding enzymes in histone modification, and mutations in the histone demethylase *UTX *[37]. However, collectively these mutations are thought to occur in less than 15% of clear cell RCC, and most recently, truncating mutations in the chromatin remodelling complex gene *PBRM1 *were found in 41% (92/227) of clear cell RCC cases [38]. Data suggest that *PBRM1 *is the second major tumour suppressor cancer gene associated with clear cell RCC after *VHL*, regulating pathways associated with chromosomal instability and cellular proliferation. Notably, *VHL*, *PBRM1 *and *SETD2 *genes all map to chromosome 3p and it is speculated that physical linkage and possibly interaction of these three genes are the key drivers for the 3p loss of heterozygosity commonly seen in clear cell RCC.

### Papillary RCC

Papillary RCC (pRCC) comprises approximately 15% of malignant kidney tumours. This subtype is further classifed into types 1 and 2, which are morphologically and biologically distinct [39]. Most of these (60-70%) are type I papillary tumours, which are generally considered to be low grade and are more frequently multifocal [40]. Hereditary papillary renal carcinoma (HRPC) is associated with activating mutations in the *MET *gene on chromosome 7 [41]; this oncogene encodes a membrane tyrosine kinase receptor whose ligand is hepatocyte growth factor (HGF). Multiple downstream signalling pathways of the receptor, including P13K, influence cell proliferation, survival and mortality [41,42], and constitutive activation of the network may therefore lead to carcinogenesis. The mutation in *MET *has also been identified in a small proportion of patients with sporadic type I pRCC [43], but inactivation may occur via epigenetic mechanisms with much higher frequency [44].

Type 2 pRCC, accounting for 30-40% of papillary tumours, also occurs in hereditary and sporadic forms. However, in contrast to Type 1 pRCCs, these tumours are more likely to be high grade and cytogenetically complex, and are therefore associated with a poor prognosis. Patients with hereditary leiomyomatosis and RCC (HLRCC) have a mutation in the gene encoding the Krebs cycle enzyme fumarate hydratase (*FH*), and are at risk of developing cutaneous and uterine leiomyomas and an aggressive phenotype of type 2 pRCC [45]. Another familial form of RCC results from germline mutation of succinate dehydrogenase B (*SHDB*) [46]; *FH *and *SHDB *appear to be tumour suppressor genes in which mutations cause increased levels of Krebs cycle enzymes fumarate and succinate respectively. Similar to the pathogenic role of mutations in the isocitrate dehydrogenase metabolic enzymes (*IDH1 *and *IDH2*) in some gliomas, these mutant metabolic enzymes inhibit prolylhydroxylases in the cell cytosol, which leads to stabilisation of HIF1-alpha and activation [47-49]. Dysregulation of other cell signalling pathways, such as the MYC pathway, may also be relevant in high grade, aggressive type 2 pRCC [50].

### Chromophobe RCC

Five to ten percent of RCCs are chromophobe renal cell carcinomas (ChRCC), which can be difficult to distinguish histologically from renal oncocytoma, a benign neoplasm. However, ChRCC is usually associated with complex loss of multiple chromosomes as detected by FISH [51] and newer gene expression profiling techniques have identified the genes *CD82 *and *S100A1 *and *AQP6 *which are expressed differentially on ChRCC and oncocytomas respectively [52]. Several studies have reported upregulation of *KIT*, a membrane receptor tyrosine kinase, and have suggested that overexpression may play a pathogenic role in ChRCC [52-54] but its therapeutic relevance is not yet certain.

### Rare subtypes

The remaining RCC subtypes are rare, and less is known of their molecular characteristics. Collecting duct carcinomas, despite being of the same embryological origin as the ureter, do not necessarily share the same molecular abnormalities that characterise urothelial carcinomas. Loss of chromosome 8p in collecting duct tumours has been identified and may be associated with a worse prognosis [55]. The genetic anomalies seen in mucinous tubular and spindle cell carcinoma (MTSCC), a low grade tumour, are heterogenous and require further investigation [56,57]. Xp11 translocation tumours were first included in the WHO classification in 2004, and usually affect children and adolescents [58,59]. These tumours have a unique histological appearance and are characterised by a breakpoint at chromosome Xp11 and gene fusions between *TFE3 *transcription factor and multiple genes [60].

## Clinical patterns in mRCC

mRCC is associated with significant inter-individual variation in clinical course. Prognosis is often influenced by the histological subtype although this is yet to be fully established. Overall, papillary and chromophobe RCC are thought to portend an improved prognosis compared to clear cell RCC [61], but there are data to suggest these tumours are more resistant to treatment and are associated with poorer survival once they have metastasised [62]. Collecting duct tumours and those with sarcomatoid features behave more aggressively than clear cell RCC, with median survivals of 11 months and less than 9 months respectively [62,63].

Furthermore, it is apparent to clinicians who treat mRCC that there is marked clinical heterogeneity even within a particular subtype. It is well recognised that a subset of patients with mRCC has an indolent disease course, and many attempts have been made to characterise this group, and to develop methods of predicting the outcome for an individual patient. However, it seems likely that this observation is the result of significant genetic heterogeneity which exists within a histopathological subtype, even when this subtype appears to be dominated by mutations in a single gene. This heterogeneity was clearly demonstrated in the earlier described gene sequencing studies in clear cell RCC [37].

The model most widely used to predict clinical outcomes in mRCC is that defined by the Memorial Sloan Kettering Cancer Center (MSKCC) [9]. A retrospective analysis grouped patients treated in clinical trials of cytokines into favourable, intermediate and poor risk categories on the basis of five factors predictive of short survival; interval from diagnosis to treatment of less than 1 year, Karnofsky performance status less than 80%, elevated serum lactate dehydrogenase (LDH), hypercalcaemia and anaemia. This model was validated in an independent cohort of treatment-naive patients, and prior radiotherapy and sites of metastasis were also identified as negative prognostic factors [64]. Several models have been described for use in patients treated with tyrosine kinase inhibitors. Patient outcomes from the phase III, landmark trial of sunitinib versus interferon were analysed and 11 pre-treatment factors were used to develop a nomogram which predicts the probability of 12 month progression free survival [65]. This tool is clearly restricted to those treated with sunitinib and may not be generalisable to patients outside a clinical trial; it also requires independent validation. Heng et al. reported that components of the MSKCC model are valid in patients treated with VEGF-targeted therapy (anaemia, hypercalcaemia, Karnofsky performance status and time from diagnosis to treatment of less than 1 year) and added platelet and neutrophil counts as independent adverse prognostic factors [66]. This analysis was based on a heterogeneous patient population and the authors argue that is widely applicable to patients treated in the kinase inhibitor era. However, all of the models described above are limited on the basis of retrospective analyses and their utilisation of pre-treatment risk factors only.

Prognostic models such as these have been used to select those patients with an improved risk profile who may benefit from alternatives to systemic therapy. For example, it is common clinical practice to observe patients with indolent, asymptomatic metastatic disease and a recent retrospective review confirmed that this strategy may be reasonable for some patients, although the selection criteria are yet to be defined, and prospective confirmatory data are required [67]. Surgical resection of metastatic disease is another therapeutic option, particularly historically when there was a lack of effective systemic therapy. Anecdotal reports of metastasectomy resulting in long term survival date back to 1939 [68], and 5 year survival rates associated with complete resection of metastases ranged between 35 and 60% [69]. Recently, Alt et al. reported on the survival of 887 patients treated with nephrectomy, who subsequently developed metastases [70]. Of these, 124 patients underwent surgical resection of all metastases and had a median 5-year cancer specific survival rate of 32.5%, compared to 12.4% for those patients who did not undergo metastasectomy. Outcomes were further improved in patients with lung-only metastases, who had a 5-year cancer specific survival rate of 73.6% if completely resected. There are also recent reports of patients achieving durable complete responses through a combination of systemic anti-angiogenic therapy and subsequent metastasectomy [71,72]. Surgical series of patients with mRCC are small with a heavy selection bias; nevertheless, when complete histological resection is achieved, metastasectomy for some individuals anecdotally improves survival, and may be curative in a minority. Surgery to remove metastases may also provide effective symptom palliation, and in the setting of oligo-metastastic disease, it might obviate the need for systemic treatment, which is usually associated with side effects. Again, there is no robust evidence to guide selection of patients for surgery, and the optimal timing of surgery and integration of treatment modalities in the targeted therapy era are unknown.

## Systemic therapy for mRCC and personalised medicine

### Systemic treatments used in mRCC

As discussed previously, cytotoxic and hormonal therapies have had a limited role in the treatment of mRCC, and until 2005, immunotherapy was the treatment of choice, despite providing clinical benefit for only a minority of patients. Since 2005, a number of new agents have been developed and approved for use in this disease with substantial improvements in patient outcomes. Broadly, current treatments can be classified as anti-VEGF agents, mTOR inhibitors, immunotherapy and cytotoxics.

Sunitinib is an oral inhibitor of multiple receptor tyrosine kinases (RTKs), including VEGFR, PDGFR, FLT-3 and c-KIT [73,74]. The phase III trial of sunitinib versus interferon, in patients with untreated, clear cell mRCC, established sunitinib as the standard of care for first-line treatment, with a median progression free survival time of 11 months for patients treated with sunitinib, compared with 5 months for those treated with interferon. A statistically significant difference in overall survival was found when patients who crossed over from interferon to sunitinib (approximately 1/3 of those randomised) were excluded from the analysis [75]. These results were confirmed in a broader mRCC population in an expanded access trial [76]. Sorafenib is also an orally administered multi-targeted tyrosine kinase inhibitor (TKI), inhibiting VEGFR-2 and -3, PDGFR, FLT3, cKIT, RET, BRAF and CRAF [77], and also resulted in an approximate doubling of median progression free survival times compared with placebo in patients who had progressed after first line cytokine therapy; median PFS for sorafenib was 5.5 months compared with 2.8 months in the placebo group [78]. Again, overall survival results were confounded by patient crossover from placebo to sorafenib. A third drug in this class is pazopanib, a second generation TKI targeting VEGFR-1, -2 and -3, PDGFR and cKIT; in particular, preclinical studies established it as very potent inhibitor of VEGFR-2 [79]. Based on a randomised phase III trial, pazopanib appears to have similar efficacy to sunitinib in the first line treatment of mRCC, with a median PFS of 9.2 months, compared to 4.2 months for placebo-treated patients [80], and an overall survival difference of 2.4 months in favour of pazopanib [81]. Results of a phase III trial directly comparing sunitinib and pazopanib are awaited.

**Figure 1:**
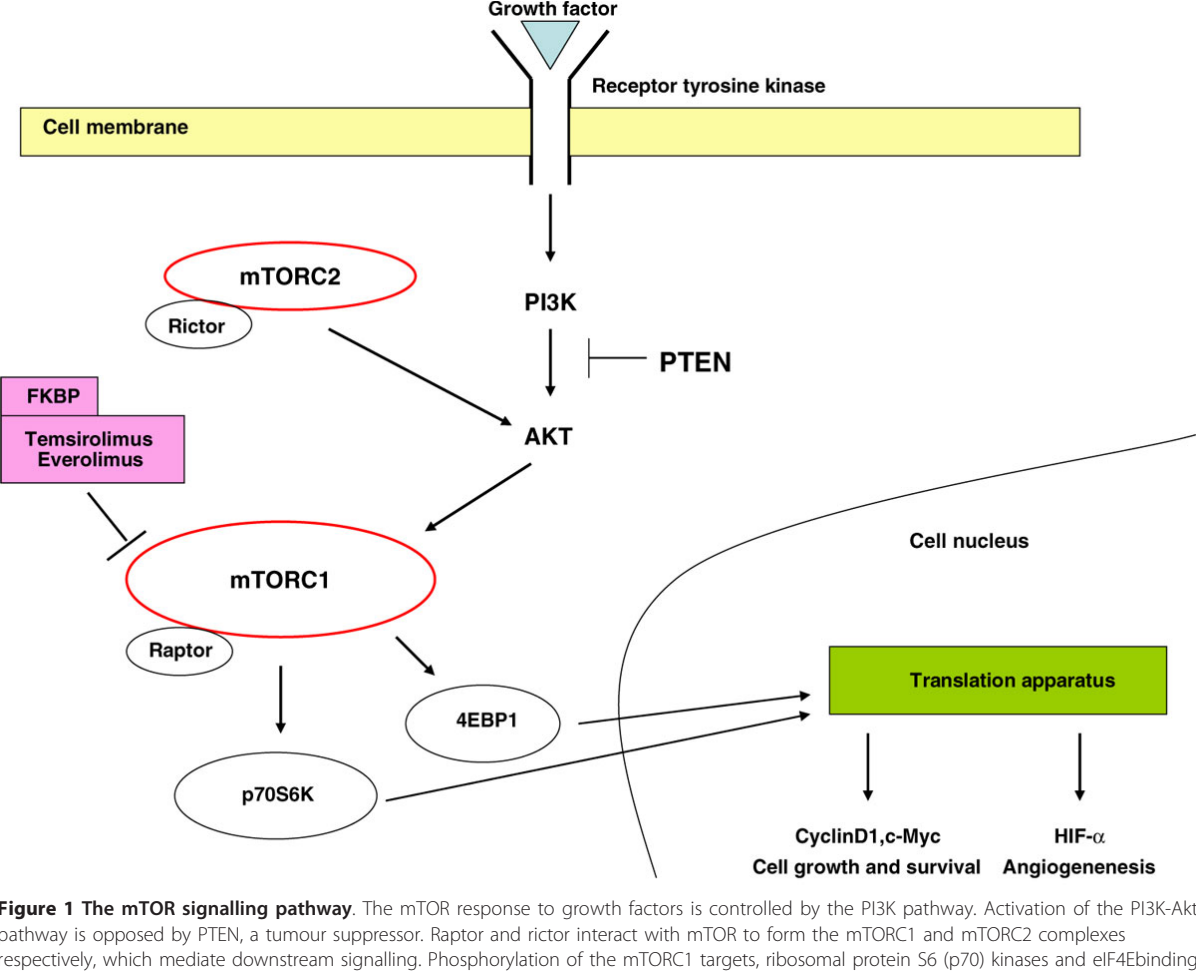
**The mTOR signalling pathway**. The mTOR response to growth factors is controlled by the PI3K pathway. Activation of the PI3K-Akt pathway is opposed by PTEN, a tumour suppressor. Raptor and rictor interact with mTOR to form the mTORC1 and mTORC2 complexes respectively, which mediate downstream signalling. Phosphorylation of the mTORC1 targets, ribosomal protein S6 (p70) kinases and eIF4Ebinding proteins (4E-BPs), results in promotion of mRNA translation, stimulation of protein synthesis and entry into the G1 phase of the cell cycle. The rapamycin-insensitive mTORC2 complex functions to regulate Akt and the cytoskeleton.

The mammalian target of rapamycin (mTOR) is a non-receptor tyrosine kinase in the PI3K-Akt pathway. Activation of mTOR has multiple downstream consequences including upregulated HIF expression [31] and effects on intracellular signalling pathways involved in cell growth and proliferation [82,83]. The mTOR inhibitors, temsirolimus and everolimus, bind to FK506-binding protein (FKBP) and this protein-drug complex inhibits the kinase activity of the mTOR complex 1 (mTORC1) [84]. Disruption of mTOR signalling results in reduced translation of cell cycle regulatory proteins, such as D-type cyclins, c-myc and p27^kip-1 ^[85], and suppressed angiogenesis [86,87] (Figure 1). Temsirolimus, an intravenously administered mTOR inhibitor, significantly prolonged median overall survival in patients with untreated, poor-risk mRCC, when compared with interferon, or the combination of inter-feron and temsirolimus in a phase III trial (10.9 months for temsirolimus, 7.3 months for interferon, and 8.4 months for the combination) [88]. Everolimus is an oral mTOR inhibitor and was established as the standard of care for second line treatment after failure of VEGF-directed TKI therapy following a phase III trial in which everolimus treatment resulted in significantly prolonged progression free survival compared to placebo [89].

Bevacizumab is a humanised monoclonal antibody against VEGF. A randomised phase II trial established that it had clinically meaningful activity in patients with mRCC [90] and two independent phase III trials combined bevacizumab with interferon-α in an attempt to improve its efficacy [91,92]. Both studies reported improved progression free survival with combination treatment compared to interferon alone, and justify the use of this combination in first line therapy of metastatic RCC in intermediate- and good-risk patients. However, neither trial observed a difference in overall survival from the addition of bevacizumab, possibly because the majority of patients in both studies received subsequent, second-line therapy which included VEGF-targeted treatments.

**Figure 2:**
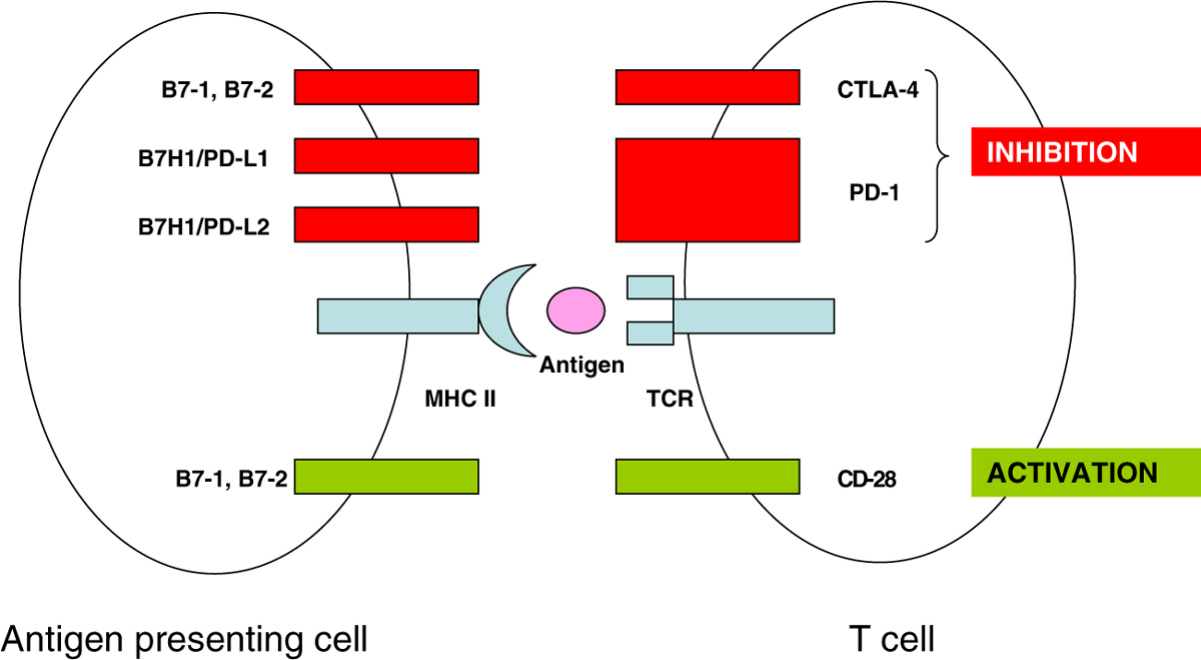
**Model of immune checkpoint molecules and T cell function**. Inhibitory T cell co-receptors such as CTLA-4 and PD-1 cause T cell anergy and apoptosis, impeding anti-tumour immunity. Abbreviations: *B7H1/PD-L1 and B7H1/PD-L2 *ligands for PD-1; *PD-1 *programmed death-1; *MHC II *major histocompatability complex class II.

Immunotherapy and cytotoxic agents are no longer considered standard therapies in mRCC. However, the regimen of capecitabine and gemcitabine chemotherapy has demonstrated activity in mRCC [93-96], and there is a recent report of two patients with prolonged remissions after treatment with this combination, and in the setting of failed targeted therapy [97]. There are also encouraging early results from a novel immunotherapy, anti-PD-1 (MDX-1106), which acts to enhance anti-tumour T-cell response [98]. PD-1 is a so called 'immune checkpoint molecule' found on activated T, B and myeloid cells, whose ligand is B7-H1--the interaction between PD-1 and B7-H1 down-regulates T cell activation [99] (Figure 2). B7-H1 is constitutively expressed in many human carcinomas but not in normal tissues [100] and expression of B7-H1 in nephrectomy specimens has been associated with a worse prognosis in patients with RCC [101].

It is important to note that patients with non-clear cell mRCC have been largely excluded from the pivotal clinical trials of the newer agents, apart from the randomised study of temsirolimus [88] and the expanded access trials of sunitinib and sorafenib [76,102]. The largest retrospective series of patients with papillary or chromophobe mRCC treated with sunitinib or sorafenib demonstrated that there was clinically meaningful activity of these agents for some individuals, but that this was lower than that observed in patients with clear cell mRCC [103]. Subset analyses of the phase III temsirolimus trials suggest greater benefit of temsirolimus (relative to interferon) in patients with non-clear cell mRCC [104,105]. A prospective phase II trial examining the response rate to sunitinib in papillary mRCC found it was significantly lower in these patients compared with clear cell patients [106], but further data on the efficacy of anti-VEGF and mTOR treatments in non-clear cell histologies from phase III trials are awaited.

### Personalised systemic therapy in mRCC

Despite clear advances in the treatment of mRCC with the development of the above-mentioned drugs, molecularly directed personalised medicine approaches for this condition have not been achieved. There are no established criteria by which to select one treatment over another for an individual patient, apart from clinical factors such as the desire to have oral rather than intravenous therapy, and patient co-morbidities and performance status. Equally important is the lack of predictors of response or resistance to therapy. This is in obvious contrast to other tumour types, such as lung, breast, and colorectal cancers, and now melanoma, in which biomarkers are used routinely to predict response to treatment. Biomarkers can be defined as molecular, cellular or functional measurable parameters indicative of important clinical events such as cancer onset, recurrence, progression or death [107]. The fact that none have been identified for use in mRCC may be because agents such as sunitinib act on non-tumour components such as the endothelium, rather than on the tumour cell itself [108], but may also reflect a lack of systematic tissue collection in the drug development trials for mRCC to date. None of the phase III trials of the drugs currently in use for mRCC included mandatory tissue collection in their protocols, and no prospective translational endpoints were included in these studies. Another issue unique to RCC and which impedes the identification of biomarkers is its marked tissue heterogeneity. Biomarker discovery trials to date have been based on the assumption that a single biopsy reflects the somatic mutation landscape of the tumour, but variation of growth pattern and cytological features are frequently identified within the same lesion and sampling of multiple areas within the same lesion would be required to assess the extent of molecular heterogeneity [109].

#### Candidate biomarkers in RCC

Research into potential biomarkers for mRCC has focussed on the clear cell subtype, because it is the dominant histological type, but also because the molecular pathogenesis has been well defined. In almost all cases of clear cell RCC there is loss of function of the *VHL *tumour suppressor gene, yet *VHL *gene status has not been reliably demonstrated to have prognostic or predictive value. Choueiri et al. demonstrated a differential response rate in 123 patients with clear cell mRCC treated with anti-VEGF therapy (sunitinib, sorafenib, axitinib or bevacizumab) according to *VHL *status [110]. Those patients with inactivated *VHL*, defined by mutation or methylation, had a similar response rate to those with wild-type VHL (41%, compared to 31%). Those with a loss of function mutation in VHL had a higher response rate of 52%, but this did not translate into improved progression-free or overall survival. Obvious limitations of this analysis are its retrospective nature and differences in the treatments given, and the overall survival data were immature at the time of publication. The improved response rates were not confirmed by two further studies; in 13 patients treated with axitinib, and in 78 patients treated with pazopanib, there was no relationship between VHL gene status and response [111,112].

HIF plays a critical role in the VHL pathway as a transcription factor for angiogenic genes in the absence of functional VHL protein. It consists of two alpha subunits (HIF-1 alpha and HIF-2 alpha) and one stably expressed beta subunit. The two alpha subunits are thought to have similar but not fully redundant functions, and it has been suggested that HIF-2-alpha may be more important than HIF-1-alpha in angiogenesis [113]. Gordan et al. distinguished two types of *VHL*-deficient clear cell RCC; those that express HIF-1-alpha and those that do not [114], but the clinical relevance of this remains to be determined and there have been conflicting results from studies examining HIF-1-alpha expression as a prognostic biomarker [115-118]. In a retrospective study of archival tissue from 43 patients with clear cell mRCC treated with sunitinib, high pre-treatment levels of HIF-1-alpha or HIF-2-alpha predicted sensitivity to treatment but these results require prospective validation [119].

Two of the many HIF-responsive genes, *VEGF *and *CAIX*, have also been explored as potential biomarkers in mRCC. The VEGF family is a key mediator of angiogenesis and comprises multiple VEGF ligands, and three cognate tyrosine kinase receptors. In the phase III trial comparing sorafenib with placebo (TARGET trial), baseline plasma levels of VEGF were available for 712 patients. Retrospective exploratory analysis revealed that high baseline VEGF levels were associated with reduced overall survival, but there was no relationship demonstrated between changes in VEGF and soluble VEGF receptor 2 (sVEGFR-2) levels during treatment and patient outcomes [120]. Pre-treatment serum VEGF levels were also shown to be prognostic in a study of 302 patients with mRCC enrolled in cytokine clinical trials [121], but were not predictive of response to bevacizumab in patients treated in the AVOREN study [122]. However, Rini et al. demonstrated that baseline plasma soluble VEGF receptor 3 (sVEGFR-3) and VEGF-C levels correlated with improved response rates and longer progression-free survival times in patients treated with suni-tinib [123]. Two small phase II studies of sunitinib and pazopanib in mRCC also suggest a potential role for VEGF and the soluble forms of the VEGF receptor as predictive biomarkers; the first study found that significantly larger reductions in VEGF, sVEGFR-2 and sVEGFR-3 levels occurred in patients exhibiting an objective response to sunitinib, and in the second, decreases in sVEGFR-2 were significantly correlated with tumour response [112,124]. Given these conflicting results, the clinical utility of the VEGF family as predictive biomarkers is uncertain.

Similarly, studies evaluating the potential of *CAIX *as a biomarker in mRCC are hypothesis-generating only. *CAIX *is a surface transmembrane enzyme that is highly expressed in advanced RCC but not in normal kidney tissue [125]. In several studies, tumours with low *CAIX *staining by immunohistochemistry were associated with worse clinical outcomes [125,126], but the largest series which examined 730 clear cell RCC specimens, reported by Leibovich et al., did not validate these findings [127]. There is limited evidence from two small studies to suggest that high tumour *CAIX *staining may be a useful predictor of benefit from interleukin-2 immunotherapy [128,129] and is currently under evaluation by the Cytokine Working Group in the SELECT trial. *CAIX *does not appear to be predictive of response to sunitinib, sorafenib or temsirolimus [120,130].

Neutrophil gelatinase-associated lipocalin (NGAL) has shown promise as predictive biomarker for mRCC patients treated with sunitinib. NGAL is a protein that is present in low levels in some human tissues, but is induced by epithelial damage in the kidney, colon, liver and lung [131]. It is a marker of acute and chronic kidney injury [132,133] and is elevated in a number of cancers, when it is usually associated with a poor prognosis [134]. It may be complexed with matrix metalloproteinase-9, which plays a role in invasion and metastasis. Porta et al. compared the predictive value of the Motzer scoring system with baseline serum concentrations of VEGF and NGAL in a cohort of 85 patients treated with sunitinib [135]. Both biomarkers correlated significantly with progression-free survival, whereas MSKCC score did not. Patients classified as having high levels of VEGF or NGAL (according to a threshold defined by the manufacturer of the ELISAs used) had a relative risk of progressing of 2.14 and 1.86 respectively, compared to patients with normal levels.

Components of the mTOR pathway have been evaluated as potential biomarkers in mRCC. Constitutive activation of the mTOR kinase via the PI3K and AKT signalling network results in activation of substrates, such as the ribosomal subunit S6 kinase (S6K) and eukaryotic initation factor 4E, which are critical for synthesis of proteins involved in cellular growth and survival. Activation of the AKT pathway can occur through homozygous loss of the *PTEN *tumour suppressor gene, but there is no definite prognostic significance for *PTEN *loss in mRCC [136,137], and there does not appear to be a correlation between tumour *PTEN *expression and benefit from temsirolimus [138]. There are also conflicting results for phosphorylated AKT (pAKT) as a prognostic biomarker [136,137]. However, Cho et al. found a positive association between phosphorylated S6 (pS6) expression and a trend towards positive expression of pAKT with response to temsirolimus [130]. Elevated lactate dehydrogenase (LDH) also appears to predict for an increase in overall survival with temsirolimus in poor-risk patients treated with temsirolimus [139]. LDH is a serum enzyme, which is regulated by the PI3K-Akt-mTOR pathway and tumour hypoxia. It is elevated in many cancers, and has prognostic significance in RCC. In this study of 404 patients treated with temsirolimus or interferon, there was a significant increase in patients with an elevated LDH treated with temsirolimus (*n *= 140), but in patients with a normal LDH (*n *= 264) there was no survival difference between temsirolimus and interferon treatments.

There is an emerging role for genetic biomarkers in prognostication in mRCC. Traditional cytogenetic karyotyping studies have demonstrated loss of 3p, 4p, 9p and 14q as possible prognostic genetic biomarkers in mRCC [140], but modern techniques such as gene expression profiling and single nucleotide polymorphism (SNP) genotyping seem more likely to be able to pinpoint specific carcinogenetic events or predict outcomes from treatment. Two studies analysing gene expression profiles in clear cell RCC have identified panels of candidate genes that appear to correlate strongly with survival and recurrence-free interval [141,142]. The first study of 177 clear cell tumours classified tumours into two subsets, defined by a set of gene features, each with distinct prognoses and biological behaviours. The second study is the largest genomic series to date, and analysed 931 archival specimens from the Cleveland Clinic by RT-PCR for expression of 727 genes (including 5 reference genes). Sixteen genes were identified as strongly associated with recurrence-free interval, and interestingly, among these 16 genes, increased expression of angiogenesis and immune-related genes correlated with a lower risk of recurrence, whereas a higher risk of recurrence was demonstrated with expression of genes associated with epithelial to mesenchymal transition. It is hoped that validation of results achieved in studies such as these may lead to the development of multiple gene algorithisms, which could be used to predict recurrence of RCC.

There is preliminary evidence that SNP genotyping could be used to recognise SNP variants that influence prognosis in patients with mRCC. In a study of 80 patients with mRCC, analysis of 21 NSPs within 13 cytokine genes revealed that heterozygosity for the IL-4 genotype -589C-33C resulted in a five-fold reduction in median overall survival, compared with homozygotes for IL-4 haplotype -589C-33C [143]. Another study identified three SNP polymorphisms in the VEGF gene associated with survival [144], and in pazopanib-treated patients, SNPs in genes related to angiogenesis, and pazopanib mechanism of action and metabolism have been associated with overall survival [145].

Finally, there are clinical and functional parameters that may prove to be useful tools in predicting patient outcomes and response to targeted treatments in mRCC. Although retrospective, there is rather compelling data demonstrating a consistent relationship between hypertension induced by anti-VEGF treatments, and improved clinical outcomes. In a retrospective pooled analysis of four studies of sunitinib in mRCC, patients who achieved a maximum systolic blood pressure of 140 mmHg or higher had marked improvements in objective response rates, progression-free and overall survival, and a weaker association was observed in sunitinib-induced hypertension when defined by a maximum diastolic blood pressure [146]. Similar results have been reported in patients treated with axitinib and bevacizumab [147,148]. There are obvious limitations to these retrospective analyses, and a prospective study of first-line axitinib (a newer multi-targeted TKI) will evaluate the strategy of dose escalation of axitinib in the absence of treatment-related hypertension or toxicity, on the basis of a study demonstrating that hypertension and axitinib drug levels are independently associated with clinical outcome [149]. Furthermore, SNPs of VEGF and VEGFR that predict the occurrence of hypertension in patients receiving sunitinib have been proposed [150].

Early, small studies of treatment-induced radiographic phenomena have shown promise but require much larger, prospective investigation. For example, changes in tumour blood flow as measured by arterial spin labelling magnetic resonance imaging (MRI) after 1 month of treatment with PTK787, a small molecule VEGF inhibitor, correlated with objective response at 4 months of therapy [151]. An alternative functional MRI technique found that the baseline volume transfer constant of the contrast agent (Ktrans), indicating higher vascular permeability, correlated with subsequent progression free survival on sorafenib, but changes in Ktrans during treatment did not predict for clinical outcome [152].

In summary, there are no biomarkers that have been approved for use in mRCC. Although the data described above provides many promising leads, it is limited by retrospective analyses, and inconsistencies in research-based assays, which in turn depend on the method of sample collection, processing and interpretation [131].

#### Drug resistance and molecular heterogeneity also impede PPPM

Other challenges faced by clinicians and scientists in the pursuit of personalised medicine for mRCC include resistance to therapy and intra-tumoural heterogeneity. Between a third and two-thirds of patients with mRCC have tumours refractory to anti-VEGF and mTOR inhibitor treatment from the outset, and all patients inevitably acquire resistance to therapy [153]. There is an increasing understanding of mechanisms of resistance, which may include vascular resistance through activation of alternative pro-angiogenic pathways [154], and hypoxia resistance [153]. Resistance to mTOR inhibition is less well understood but preclinical models suggest that negative feedback loops and parallel signalling pathways such as the Ras-mitogen-activated protein kinase (MAPK) pathway play an important role [153]. Again, routine tissue collection at important clinical time-points (such as pre-treatment, and at the time of progressive disease) is likely to add to the mounting body of evidence delineating resistance pathways. The European Union multi-disciplinary Personalised RNA Interference to Enhance the Delivery of Individualised Cytotoxic and Targeted therapeutics (PREDICT) consortium has identified four key areas of research, and obstacles to individualised therapy in mRCC--these are the identification of predictive and surrogate biomarkers, determination of the mechanisms of resistance and response to VEGF-targeted therapy, and the identification of new targets [155]. It is hoped that these endpoints will be addressed through the analysis of tumour tissue collected in pre-operative biopsy studies in mRCC, using novel methods of personalise tumour-derived small hairpin RNA and high-throughput small interfering RNA screens.

## Intratumour heterogeneity: a challenge to personalised medicine and biomarker discovery

It is clear that the histological subtypes of RCC differ in their molecular profiles, and systematic screens of genes involved in RCC have revealed substantial genetic heterogeneity even within the clear cell subtype, previously thought to be dominated by a mutation in a single gene, *VHL *[37,156,157]. Furthermore, it is emerging that intratumour heterogeneity exists. It is this which presents the biggest challenge to biomarker discovery in RCC, and genetic instability may independently contribute to multiple drug resistance [158,159].

Moch et al. analysed 53 clear cell and papillary RCCs by fluorescence in situ hybridisation (FISH) for *VHL *deletions, and confirmed that *VHL *deletions were present in 69% of clear cell RCCs, consistent with previous studies. However, within individual clear cell tumours, subpopulations of cells with and without *VHL *deletion existed, and *VHL*-depleted subpopula-tions had different chromosome 3 counts [157]. The situation has become much more complex since this study was published, with the discovery of multiple genes implicated in RCC, and there are intensive efforts underway to define the clonal architecture of RCC tumours. This will require multiple biopsies of individual primary and secondary tumours; to date, there are no published data using this approach in mRCC but it is a method being utilised in the PREDICT consortium's pre-operative trials of mTOR and kinase inhibitors [155].

## Conclusions and outlook

There have been significant developments in the treatment of mRCC in recent years, but preventive, predictive and personalised medicine for this condition remains elusive. Failure to provide such tailored treatment for patients clearly has profound clinical and social implications for individuals but also impacts heavily on health economies. Personalised medicine will not be achieved unless there is an improved understanding of the mechanisms which underpin response and resistance to therapy, and biological markers of these processes. A paradigm shift is required, in which clinicians select treatments based on molecular, rather than anatomical or histopathological criteria. After all, knowledge of molecular pathways in RCC has led to the development of the therapeutic agents which have already resulted in vast improvements in clinical outcomes for patients. However, enhanced understanding of comprehensive molecular networks, and intra- and inter-tumour heterogeneity is the key to further improvements in the treatment of mRCC. Clinical trial design will need to adapt in order to achieve this goal; in particular, pre-operative studies in this field offer a unique opportunity for researching the biology of this complex malignancy.

Finally, this review has focussed on treatment of metastatic RCC, and whilst it is clearly desirable to improve survival and quality of life for those patients with advanced disease, it is also true that overall improvements in outcomes in this condition, as in other tumour types, depends on superior treatment of early disease and prevention of metastastic spread. Currently no standard adjuvant therapy exists for patients after nephrectomy for RCC, but there is no doubt that the progress made in the field of metastatic RCC will translate into more rational management of localised disease with appropriate integration of surgical and medical therapy.
